# Nitrogen Removal Performance and Metabolic Pathways Analysis of a Novel Aerobic Denitrifying Halotolerant *Pseudomonas balearica* Strain RAD-17

**DOI:** 10.3390/microorganisms8010072

**Published:** 2020-01-02

**Authors:** Yunjie Ruan, Mohammad J. Taherzadeh, Dedong Kong, Huifeng Lu, Heping Zhao, Xiangyang Xu, Yu Liu, Lei Cai

**Affiliations:** 1 Institute of Agricultural Bio-Environmental Engineering, College of Bio-systems Engineering and Food Science, Zhejiang University, Hangzhou 310058, China; ruanyj@zju.edu.cn; 2 Academy of Rural Development, Zhejiang University, Hangzhou 310058, China; 3 Swedish Centre for Resource Recovery, University of Borås, 50190 Borås, Sweden; mohammad.taherzadeh@hb.se; 4 Agricultural Experiment Station, Zhejiang University, Hangzhou 310058, China; ntzx@zju.edu.cn; 5 Department of Environmental Engineering, Zhejiang University, Hangzhou 310058, China; luhuifeng@zju.edu.cn (H.L.); zhaohp@zju.edu.cn (H.Z.); xuxy@zju.edu.cn (X.X.); 6 College of Life Science, Zhengjiang University, Hangzhou 310058, China; Liuyu_zju@zju.edu.cn; 7 Laboratory of Microbial Resources, College of Food Science and Biotechnology, Zhejiang Gongshang University, Hangzhou 310035, China

**Keywords:** aerobic denitrification, *Pseudomonas balearica* RAD-17, nitrogen removal, metabolic pathways, bioaugmentation

## Abstract

An aerobic denitrification strain, *Pseudomonas balearica* RAD-17, was identified and showed efficient inorganic nitrogen removal ability. The average NO_3_^−^-N, NO_2_^−^-N, and total ammonium nitrogen (TAN) removal rate (>95% removal efficiency) in a batch test was 6.22 mg/(L∙h), 6.30 mg/(L∙h), and 1.56 mg/(L∙h), respectively. Meanwhile, optimal incubate conditions were obtained through single factor experiments. For nitrogen removal pathways, the transcriptional results proved that respiratory nitrate reductases encoded by *napA*, which was primarily performed in aerobic denitrification and cell assimilation, were conducted by *gluS* and *gluD* genes for ammonium metabolism. In addition, adding the strain RAD-17 into actual wastewater showed obvious higher denitrification performance than in the no inoculum group (84.22% vs. 22.54%), and the maximum cell abundance achieved 28.5 ± 4.5% in a ratio of total cell numbers. Overall, the efficient nitrogen removal performance plus strong environmental fitness makes the strain RAD-17 a potential alternative for RAS (recirculating aquaculture system) effluent treatment.

## 1. Introduction 

Biological nitrogen removal is crucial for wastewater treatment, and the heterotrophic denitrification is the most selective method due to its high efficiency and flexibility [1]. During the process, organic carbons are necessary to supply the electrons to the nitrate. Substances such as methanol, ethanol, etc., were commonly used [2]. However, precise liquid organic was difficult to measure, and an overdose can add organic loading to the bio-filter, which would have negative effects on the aquaculture system stability [3]. In wastewater with low C/N ratio characteristics, solid-phase denitrification that use biodegradable polymer as a simultaneous organic carbon source, as well as biofilm carriers, are considered more appropriate in specific fields [4]. For example, in recirculating aquaculture system (RAS) effluent, PBS (poly-butylene succinate) or PHBV (poly 3-hydroxybutyrate-co-3-hydroxyvalerate)-based denitrifying reactors demonstrated efficient nitrogen removal performance [3,5]. Therefore, using biodegradable polymer as slow-release organic will also have additional benefits to increase the fish survival rate [3,6].

The drawback of the biodegradable polymer-based denitrification is the relative high cost in media when compared with liquid carbon [4]. On the other hand, residual DOC (dissolved organic carbon) in effluent commonly appeared in solid-phase denitrifying reactors [3,7,8], which indicated that the entirety of the carbon source was not optimally used since part of degrading bacteria have an incapable ability in denitrification [9]. Moreover, the residual organic substances were considered to support DNRA (dissimilatory nitrate reduction to ammonium) over denitrification [10], as well as the SRB (sulfate reduced to sulfide) process [3], especially in marine environment conditions. Therefore, enhancement of the denitrification performance is crucial for this technology in practice.

An interesting alternative to promote the denitrification performance is the supplement of functional bacteria through bioaugmentation technology [11]. In a previous study, adding the *Diaphorobacter polyhydroxybutyrativorans* strain SL-205 enabled rapid reactor startup and improved nitrate removal performance when compared with active sludge inoculation in an anoxic solid-phase denitrification reactor [12]. This indicated that the initial microbial regulation can support relative bacterial community in reactors. Furthermore, to suppress the DNRA and SRB pathways in a marine PBS denitrification reactor, the alternant aerobic/anoxic operations instead of continuous anoxic were demonstrated to be feasible in our previous study [6]. Therefore, the bacteria screening for bioaugmentation need strong fitness in such salinity and aerobic conditions.

Traditional denitrification or the similar reduction process only appeared under anoxic conditions [13,14], as the *narG* gene encoded for the nitrate reductase is sensitive to oxygen presence, which could block the sequential energy and electron transfer under aerobic condition [15]. Recently, many aerobic denitrifying groups were found to support a potential pathway for a biological nitrogen removal process [16]. The main characteristics of these strains are that they have a gene cluster of the *napFDAGHBC* family while *napA* was responsible for synthesis of the catalytic subunit for electron delivery from NADH+ to nitrate aerobically [17]. Under aerobic or alternate aerobic/anoxic conditions, the nitrate reductase encoded by the *napA* gene, which is located in the periplasm, was primarily infiltrated by nitrate and oxygen as compared to the *narG* gene located in plasma membrane [18]. Therefore, the high activity of the *napA* gene make these aerobic denitrifying strains use nitrate over oxygen preferentially [16]. Potentially, this wide niche can reduce technological requirements in current biological nutrient treatment processes, which always cause temporal (Anoxic/Oxic, A/O process) or spatial (sequencing batch reactor, SBR process) division for different microbiota [19]. Until now, many aerobic denitrifies were reported in plenty of genera including *Pseudomonas stutzeri* YZN-001 [20], *Acinetobacter sp.* HA2 [21], *Pseudomonas stutzeri* T13 [22], *Marinobacter hydrocarbonoclasticus* RAD-2 [23], *Pseudomonas stutzeri* C3 [24], etc. In addition, several strains were also found to have aerobic ammonium removal ability, which show potential through heterotrophic nitrification or assimilation pathways [22,24,25,26].

In this study, a novel aerobic denitrifying halotolerant strain, *Pseudomonas balearica* RAD-17, was isolated for a long-term PBS-supported denitrification reactor for RAS effluent treatment, demonstrated in a previous study [6]. The 16S rRNA gene was amplified to identity the phylogenetic relationship for the isolated strain, while API 20NE (analytical profile index of Gram-negative with non-Enterobacteriaceae) was used for its physiological feature. Meanwhile, the inorganic nitrogen removal performance was also evaluated by different nitrogen sources. Moreover, the aerobic nitrogen metabolic pathways were investigated by quantifying the key denitrifying genes (*napA*, *nirS*, *norB*, and *nosZ*) and glutamic biosynthesis genes (*gluD* and *gluS*) that are potentially related with ammonium assimilation. In addition, the strain’s bioaugmentation performance was also evaluated by adding it into actual RAS effluent. To the best of our knowledge, this is the first report of a functional strain with efficient aerobic nitrogen removal ability in the *Pseudomonas balearica* species. Overall, the results might provide new insight in aerobic denitrifying microbial resources and potential alternatives for enhancing nitrate-removal performance for RAS practice. 

## 2. Materials and Methods

### 2.1. Cultured Media

The culture media used in this study were according to our previous study [23]. The LB (Luria-Bertani) media was prepared by using 5.0 g/L yeast extract, 10.0 g/L peptone, 25.0 g/L NaCl, and 1.5% (*w/v*) agar. The DM (denitrification media) was prepared in the ratio of 2.0 g/L sodium acetate, 2.0 g/L KNO_3_ (or NaNO_2_), 0.2 g/L MgSO_4_·7H_2_O, 1.0 g/L K_2_HPO_4_, and 1.0% (*v/v*) trace-element solution for aerobic denitrification performance evaluation. The HNM (heterotrophic nitrification media) was prepared as follows: 2.0 g/L sodium acetate, 0.3 g/L NH_4_Cl, 0.2 g/L MgSO_4_·7H_2_O, 6.7 g/L Na_2_HPO_4_, 1.0 g/L KH_2_PO_4_, and 1.0% (*v/v*) trace-element solution for ammonium-nitrogen removal evaluation. The trace-element solution contained 50.0 g/L EDTA, 2.2 g/L ZnSO_4_, 5.5 g/L CaCl_2_, 5.06 g/L MnCl_2_·4H_2_O, 5.0 g/L FeSO_4_·7H_2_O, 1.1 g/L (NH_4_)6Mo_7_O_2_·4H_2_O, 1.57 g/L CuSO_4_·5H_2_O, and 1.61 g/L CoCl_2_·6H_2_O. In addition, the amounts of nitrogen and carbon in DM or HNM can also change according to the experimental setting. The initial pH of all media was set to 7.2 and then autoclaved for 20 min at 121 °C.

### 2.2. Bacteria Isolation and Identification

The RAD-17 strain was screened from a long-term PBS based denitrifying reactor, which operated under alternant aerobic/anoxic conditions in our previous study [6]. The reactor influent contained around 10 mg/L NH_4_^+^-N and 150 mg/L NO_3_^−^-N and showed average TAN and nitrate removal rates of 47.35 ± 15.62 g NH_4_^+^–N·m^−3^·d^−1^) and 0.64 ± 0.14 kg NO_3_^−^-N·m^−3^·d^−1^with no obvious nitrite accumulation [6]. For screening, a 15 mL mixture of mature PBS and solutions were transferred into a 150 mL flask aseptically with 100 mL LB media for 10 days of preculture. The temperature and revolution were set at 30 °C and 150 rpm (revolutions per minute), respectively. Afterwards, the homogenized suspensions were serially diluted and plated using a DM media and incubated at 30 °C for 72 h. Then, a single colony with a pale-yellow circle was dilution-streaked onto a DM agar plate for further purification. Finally, a strain of the *Pseudomonas balearica*, named RAD-17, was isolated. The genomic DNA of the RAD-17 strain was extracted using a DNA extraction kit (TaKaRa Biotechnology Co. Ltd, Beijing, China). The 16S rRNA amplified product was sequenced by the Zhejiang Institute of Microbiology (Hangzhou, China). Phylogenetic relationships of the strain RAD-17 with other denitrifying bacteria were constructed using the molecular evolutionary genetics analysis software (MEGA 5, The Biodesign Institute, Tempe, USA). In addition, the purified strain RAD-17 was also stored in a 30% glycerol solution at −80 °C for following experiments.

### 2.3. Nitrogen Removal Performance

The inorganic nitrogen removal performance of the strain RAD-17 was evaluated on DM or HNM media. For aerobic denitrification capacity, a sole nitrogen source of NO_3_^−^-N (around 300 mg/L) or NO_2_^−^-N (around 300 mg/L) was tested in DM media, which contained KNO_3_ or NaNO_2_, respectively. For heterotrophic ammonium removal, sole nitrogen source of NH_4_Cl (around TAN 80 mg/L) was carried out in similar operation in HNM media. For process, 3% (*v/v*) seed suspension was inoculated in 250 mL Erlenmeyer flasks and cultured for 48 h under aerobic condition at 25 °C and 150 rpm, respectively. Meanwhile, the cell-growth (OD_600_ value) and nitrogen concentrations were measured every 4 h.

### 2.4. Single-Factor Experiments

Single-factor experiments were also carried out to evaluate the effect of various conditions on the aerobic denitrification performance of the strain RAD-17, for optimized incubated conditions. The basal condition was determined as follows: NO_3_^−^-N concentration of 300 mg/L, C/N ratio 10, NaCl concentration 25‰, temperature 25 °C, rotation 150 rpm, and 3% inoculation (*v/v*). On C/N ratio test, the C/N ratios were set to 2, 5, 10, 15, and 20. On salinity test, the NaCl concentrations were set at 0‰, 2.5‰, 5‰, 15‰, and 25‰. On carbon sources, fructose, sodium acetate, lactin, glucose, and sodium citrate were tested. On revolution test, the speeds were set to 0 rpm, 50 rpm, 100 rpm, 150 rpm, and 200 rpm. On temperature test, 5 °C, 15 °C, 25 °C, and 40 °C were used. All tests were conducted in triplicate and non-seeded samples were used as blank control.

### 2.5. qRT-PCR Analysis 

The transcriptional level gene expression intensity of the strain RAD-17 on nitrogen removal processes was investigated to reveal the metabolic pathways. In this study, real-time quantitative PCR was conducted to amplify the denitrifying genes *napA*, *nirS*, *norB*, *nosZ*, and the ammonium incorporation genes *gluD* (NADP-specific glutamate dehydrogenase) and *gluS* (glutamate synthase) with RNA samples in 48-h experiments. All primers were designed by the genome sequence of the strain RAD-17 and are listed in Table 1. The amplification specificities of these primer pairs were verified through agarose gel electrophoresis (Figure S2). The housekeeping gene 16S ribosoml RNA was used as an internal control to normalize the data. Total RNA extraction and cDNA synthesis were performed by using an RNAprep Bacteria Kit and a FastQuant RT Kit (Tian Gen BiotechCo. Ltd, Beijing, China), respectively. PCR amplification was performed with the following protocol: Initial denaturation at 95 °C for 2 min followed by 40 cycles of denaturation at 95 °C for 15 s, annealing at 55 °C for 15 s, and synthesis at 72 °C for 15 s; a melting curve was generated by linear heating from 70 °C to 95 °C over 25 min [27]. All quantitative amplifications were conducted in triplicate using the SYBR Green Real-Time PCR Kit (Novland, Shanghai, China) and respective primers on a StepOne PCR instrument (Applied Biosystems, Forest City, CA, USA).

### 2.6. Bioaugmentation Performance Evaluation

The strain RAD-17 was added into actual RAS effluent to evaluate its bioaugmentation performance. The experimental tanks had a total volume of 10 L. A total of 5 L was used for raw RAS wastewater. The NO_3_^−^-N concentration and the C/N ratio were adjusted at approximately 100 mg/L and 15 by adding KNO_3_ and sodium acetate, respectively. Then, 500 μL of the strain RAD-17 (OD_600_ = 1.0) solution was added into the experimental tanks and aerobically cultured for 140 h. Another group without inoculation operated as control. Air-pumps (ACO-003, 120 W, Sengseng Co., Ltd., Taipei, Taiwan) were used for aerobic condition and temperature was set at 25 ± 1 °C in a thermostatic chamber. All treatments were carried out in triplicate.

During the experimental phase, 2 mL solutions were cultured on DM media to evaluate the potential denitrification strains by the most probable number (MPN) standard method. Here, the amount of CFU can partly reveal the potential denitrifying ability as DM is a specific media for the denitrifier [28]. Meanwhile, the growth rate of the strain RAD-17 was also detected through monitoring the copies ratio of the *napA* gene by the strain RAD-17 that is relative to the total 16S rDNA genes using absolute qPCR. The qPCR primers are listed in Table 1. In the absolute qPCR assay, the standard curves were constructed using serial dilution of purified target DNA from PCR amplification. The amount of the template DNA was determined by the NanoDrop ND-2000 ultraviolet absorption assay. There are different gene copies between the *napA* gene and the 16S rDNA gene. The average number of 16S rDNA copies per bacterial cell is 4.2, and the *napA* gene carries a single copy in the strain RAD-17 cell [19]. The different copy numbers of two genes were used to normalize the ratio of qPCR data.

### 2.7. Analytical Methods

The solution samples were filtered through a 0.45 μm filter membrane before analyzing. The TAN, NO_2_^−^-N, and NO_3_^−^-N concentrations were analyzed according to standard methods [29]. Bacteria biomass was measured by OD_600_ value using a spectrophotometer at 600 nm (Agilent Technologies Cary 60 UV-vis, Santa Clara, USA). The morphology of the strain RAD-17 was observed by a scanning electron microscope (SEM) (SU8010, Hitachi High-Technologies Corporation, Tokyo Japan). The pH value was measured using a portable pH meter (S8, Mettler Toledo, Zurich, Switzerland). DO was measured using a DO meter (SG9-FK2, Mettler Toledo, Zurich, Switzerland). Physiological and biochemical characteristics were tested using API 20NE kits (BioMérieux Shanghai Co. Limited, Shanghai, China), and test strips were checked after incubation for 24 h [23]. Nitrogen balance analysis was done according to the previous study. For process, 3% (*v/v*) seed suspension was inoculated in 250 mL Erlenmeyer flasks and cultured for 20 h under aerobic condition at 25 °C and 150 rpm, respectively. The incubated nitrate was set at around 30 mg/L. Then, the nitrogen balance can be calculated on the initial and final nitrogen concentration [30].

## 3. Results and Discussion

### 3.1. Bacteria Characteristics and Identification

**Figure 1:**
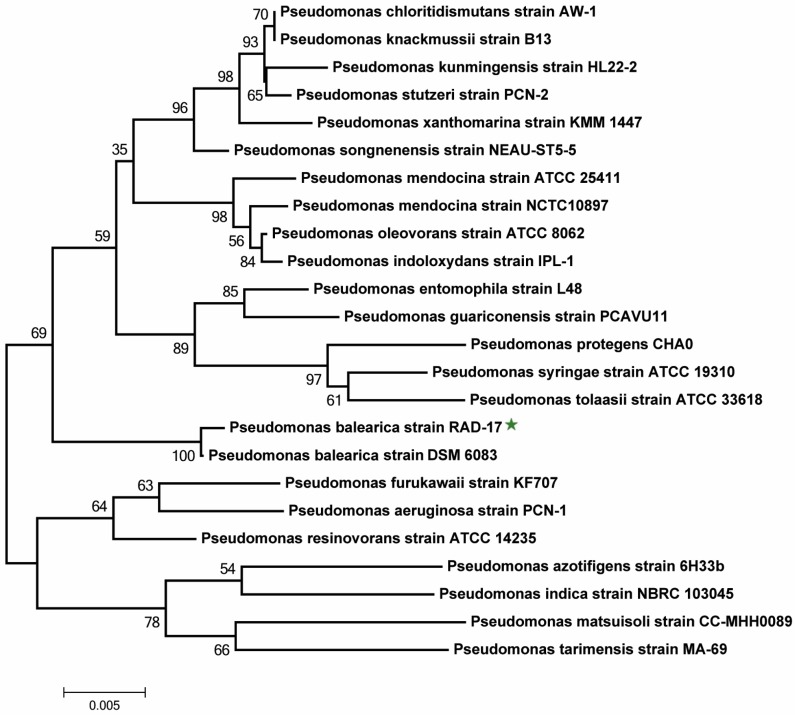
Neighbor-joining phylogenetic tree based on 16S rRNA gene sequences showing the position of the RAD-17 strain and closely related strains. Bootstrap values based on 1000 replicates are presented in branch nodes.

For strain screening, more than 20 pure isolates were obtained from the DM medium, while one named RAD-17 showed the highest aerobic denitrification performance. The colonies of the RAD-17 strain were pale yellow, salient, semitransparent, circular in shape, and presented a moist surface on the LB medium. The RAD-17 strain was a gram-negative strain with a bacilliform sharp in size of around 0.3–0.4 µm in diameter and 0.8–1.6 µm in length, respectively (Figure S1). The 16S rRNA gene sequence was submitted to the NCBI database with the accession number MK881511, and the highest similarity of the RAD-17 strain was found with *Pseudomonas balearica* DSM 6083. The phylogenetic analysis (threshold 100%) further confirmed the identification of the RAD-17 strain as *Pseudomonas balearica* (Figure 1). For nitrogen removal, many strains in the genus *Pseudomonas* were demonstrated to have aerobic denitrification ability, such as *Pseudomonas stutzeri* C3 [24], *Pseudomonas stutzeri* T13 [25], *Pseudomonas stutzeri* YZN-001 [20], *Pseudomonas stutzeri* PCN-1 [31], *Pseudomonas tolaasii* Y-11 [32], etc. In addition, several groups also have ammonium removal capacity under aerobic conditions [22]. However, in *Pseudomonas balearica*, though strain DSM6083 presented genome information (ASM81801v1), no study on the denitrifying function was reported in this sub-lineage.

The API 20NE tests were carried out for further identification of the physiological and biochemical characteristics of the strain RAD-17 (Table 2). The RAD-17 strain was positive for nitrate reduction, but was negative for urease, β-glucosidase, protease, and β-galactosidase. On cell biosynthesis, it could use glucose, maltose, gluconate, capric acid, etc., while arabinose, mannose, mannitol, and N-acetyl-glucosamine could not be assimilated. 

### 3.2. Nitrogen Removal Performance

#### 3.2.1. Nitrogen Removal Ability

**Figure 2:**
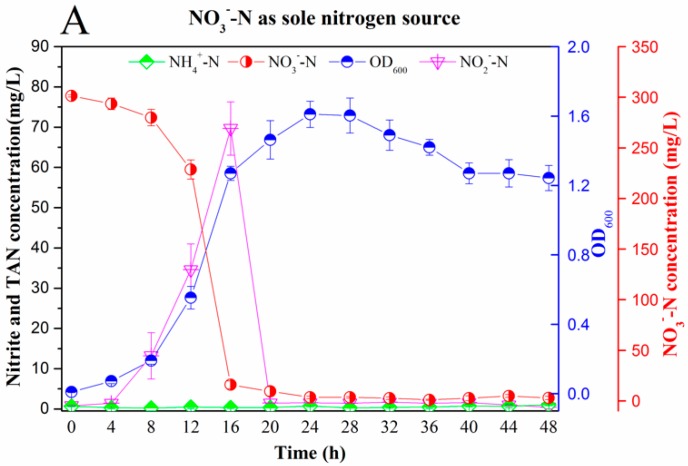

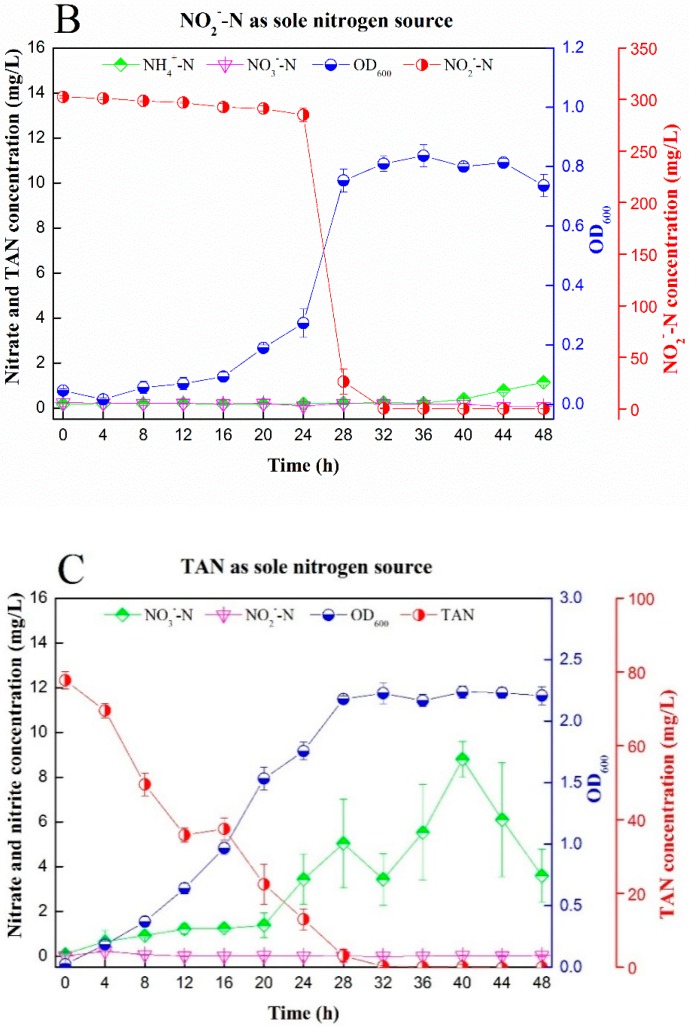
Aerobic nitrogen-removal ability and cell growth of the strain RAD-17 in denitrification media (DM) and heterotrophic nitrification media (HNM) mediums. (**A**) Nitrate as the sole nitrogen source; (**B**) nitrite as the sole nitrogen source; (**C**) ammonium as the sole nitrogen. Date shown are means ± SD (error bars) from three replicates.

The aerobic inorganic nitrogen removal capacity of the strain RAD-17 is shown in Figure 2. In general, the strain RAD-17 can use three typical nitrogen forms, such as nitrate, nitrite, and ammonia. For aerobic denitrification, nitrate as sole nitrogen showed a quick start with the NO_3_^−^-N concentration decreasing from the initial 301.43 ± 1.06 mg/L to a final 2.94 ± 1.66 mg/L, which indicated a 99.02% removal efficiency (Figure 2A). The lag phase was only observed during the initial 8 h, and the logarithmic growth phase occurred during the following 8 h. Nitrite accumulation was also presented and the peak concentration of 69.67 ± 6.64 mg/L and was found in 16 h but disappearing rapidly after 20 h. The strain biomass reached its maximum OD_600_ value with 1.61 ± 0.08 in 24 h but then slightly decreased. The reason might be that the nitrogen substance was consumed in this period. Additionally, when using nitrite as sole nitrogen, a backward lag phase was observed in 0–24 h, though the NO_2_^−^-N concentration also decreased from 302.27 ± 1.11 mg/L to 0.56 ± 0.37 mg/L rapidly (Figure 2B). The final strain biomass was around 0.74 ± 0.04, which was lower than using nitrate as the nitrogen source that potentially indicated that nitrite added toxicity to the cell. For the aerobic denitrification rate, the RAD-17 strain obtained 6.22 mg NO_3_^−^-N·L^−1^∙h^−1^ and 6.30 mg NO_2_^−^-N·L^−1^·h^−1^, respectively. The results were consistent with the previous reported in the *Pseudomonas* family, such as 7.73 mg NO_3_^−^-N·L^−1^·h^−1^ of *Pseudomonas stutzeri* YG-24 [33] and 6.52 mg NO_3_^−^-N·L^−1^·h^−1^ of *Pseudomonas sp*. ADN-42 [34].

The aerobic ammonium removal ability of the strain RAD-17 is illustrated in Figure 2C. When using ammonium as nitrogen source, no obvious lag phase occurred, indicating that ammonium should be an accessible element for cell growth. The TAN concentration decreased from the initial 77.73 ± 2.35 mg/L to the final 2.94 ± 1.66 mg/L, resulting in 1.56 mg TAN·L^−1^·h^−1^ removal rate. This phenomenon could also be supported by the higher OD_600_ value of 2.24 ± 0.05, which revealed more efficient strain yield when compared with nitrate or nitrite as substance. It was interesting to note that a slightly nitrate concentration of maximum 8.79 ± 0.80 mg/L was accumulated in 28–48 h. Under the current condition, ammonium was the sole nitrogen source, so the mechanism and transfer pathway of ammonium to nitrate and nitrogen gas still needs further study.

#### 3.2.2. Nitrogen Balance Analysis

The result of the nitrogen balance analysis is shown in Table 3. Under aerobic denitrification, the strain RAD-17 gained 87.76% nitrogen loss, which indicated that the nitrate substance prioritized transfer to gaseous products rather than biomass synthesis. Similar reports which showed 12.6% cell assimilation from nitrate were presented in *Paracoccus versutus* KS293 [35] and 19.8% in *Pseudomonas stutzeri* ZF31 [30]. On the other hand, using ammonium as sole nitrogen source, increased cell yield revealed by higher OD_600_ value was obtained (Figure 2C), which were also consisted with nearly 50% ammonium-nitrogen assimilated in *Acinetobacter sp*. HA2 and *Paracoccusversutus* LYM, respectively [21,36].

#### 3.2.3. Single Factor Experiments

The aerobic denitrification performance under different single factor conditions is shown in Table 4. In general, the C/N ratio and the kinds of carbon source should strongly associate with nitrate removal efficiency, due to this server as electron donor and energy support [13,15]. For carbon sources, the strain RAD-17 was found unable to metabolize lactin while fructose also obtained low efficiency. In contrast, more than 95% nitrate removal performance was presented in glucose, sodium acetate, and sodium citrate, respectively. In general, these substances were thought to be easily utilized as raw material for a TCA (tricarboxylic acid) cycle for maximum energy efficiency and ATP (adenosine triphosphate) synthesis under aerobic metabolism [37]. For organic amounts, a C/N ratio with a range of 5–15 was found to have optimal denitrification performance, which gained nearly complete nitrate removal. For a C/N ratio of 2, low nitrate removal performance with residual nitrite indicated electron donor deficiency. In addition, a slight decrease in nitrate removal efficiency was presented in a C/N ratio of 20, which revealed that excess organic substances also had a negative effect on denitrification. Serval similar reports were also found optimum C/N ratio 5–15 in the *Marinobacter hydrocarbonoclasticus* RAD-2 strain [23], C/N ratio 15 in *Marinobacter sp*. F6 [38], C/N ratio 7–9 in *Bacillus methylotrophicus* L7 [39], and C/N ratio 6–10 in *Pseudomonas stutzeri* YG-24 [33]. 

For salinity, the strain RAD-17 gained commendable denitrification performance in a NaCl concentration range of 0–25‰. The similar OD_600_ value and nitrate removal performance were presented in a NaCl amount of 0‰ and 25‰, which indicated that salinity has no effect on growth and the denitrifying activity of the strain RAD-17. An inferential mechanism was that Na^+^ ion supported from sodium acetate can offset the NaCl deficiency. This was also demonstrated in *Marinobacter hydrocarbonoclasticus*, as Na^+^ ion was absolutely required, no matter whether the K^+^ ion and Cl^−^ ion existed [40], and a minimal amount for its growth is a Na^+^ ion beyond 0.08 molarity concentration [41]. Therefore, the current phenomenon revealed that the strain RAD-17 might have wide ecological niche fitness in practice. 

For temperature, a typical mesophilic characteristic of the strain RAD-17 was present when more than 90% nitrate removal efficiency was found in 15–40 °C. No denitrification occurred under a temperature of 5 °C, which indicated that low a temperature might have a significant negative effect on denitrifying enzyme activity. This result was consistent with previous studies that used mostly mesophilic aerobic denitrifiers [23,42,43].

In this study, the effect of rotation speeds that revealed DO concentrations on denitrification performance were also evaluated. It should be noted that the strain RAD-17 gained both ideal nitrate removal efficiency in anoxic (0 rpm, DO 0.2 ± 0.1 mg/L) and aerobic (150 or 200 rpm, DO 3.3 ± 0.6 mg/L or 4.7 ± 0.9 mg/L ) conditions, respectively. However, a decreased denitrification performance was observed in oxygen-limited conditions (50 or 100 rpm, DO 0.9 ± 0.4 mg/L or 1.7 ± 0.8 mg/L). In a previous study, several denitrifying strains, especially in the genus *Paracoccus versutus*, have both aerobic and anoxic nitrate removal ability. For example, *Paracoccus versutus* KS293 exhibited 82% and 85% total nitrogen removal under anoxic and aerobic conditions, respectively [35]. Since denitrification is a respiratory process, the regulation of the denitrification respirome in *Paracoccus* denitrificans is related to transcription factors *fnrP*, *nnrR*, and *narR* to adopt the oxygen, nitric oxide, and nitrate shift conditions [44,45]. However, whether the *Pseudomonas* family shares similar pathways or not is still unclear. Therefore, further studies need to reveal the oxygen triggering mechanism for the strain RAD-17 denitrification in future. 

### 3.3. Nitrogen Metabolism Pathways Analysis

#### 3.3.1. Aerobic Denitrification Pathway

**Figure 3:**
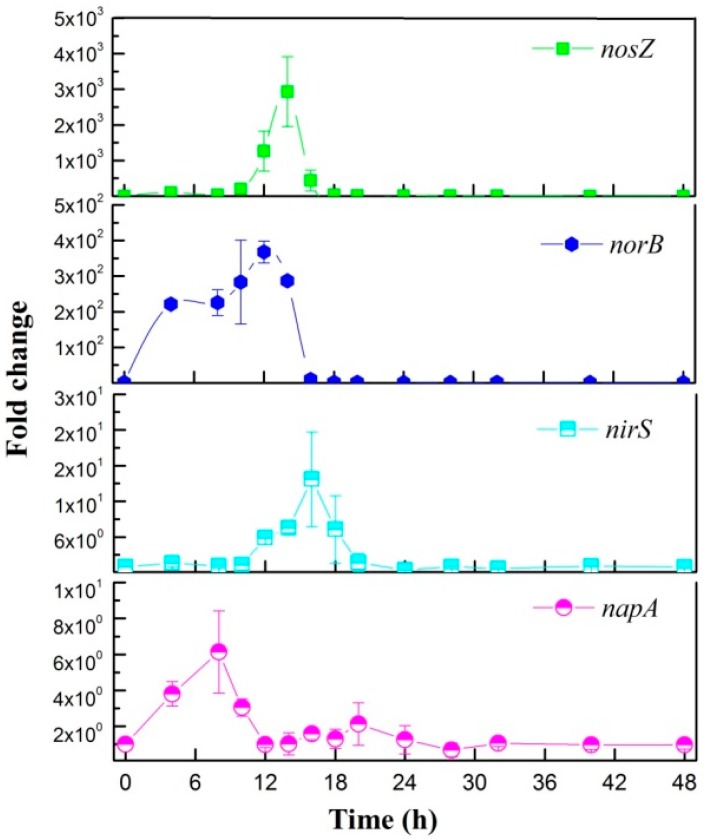
Aerobic denitrifying gene expression of the strain RAD-17 during 48 h of incubation.

The transcriptional expression levels of the denitrification genes under aerobic condition of the RAD-17 strain are shown in Figure 3. The results reveal that the expression of four respiratory nitrate reductases related genes were significantly upregulated by nitrate inducing, including *napA*, *nirS*, *norB*, and *nosZ*. The *napA* firstly showed a quicker response to nitrate than *nirS*, *norB*, and *nosZ*, which is consisted with the fact that this process is a sequence of electrons transfer [13,18]. Then, time-delay expression of *nirS* and *nosZ* were present, which was consistent with the nitrite peak concentration after 16 h (Figure 2A). It was interesting to note that the enhanced expression of *norB*, which lasts 4–16 hours incubation, presented a maximum intensity earlier than *nirS* and *nosZ*. In *Pseudomonas stutzeri* PCN-1, coincident peak expression was found in *nirS*, *norB*, and *nosZ* [31]. Therefore, these results implied that the environmental signals NO_3_^−^ and NO might both be the indirect inducer for *norB* [13]. In a previous study, the transcriptional activators, *fnrP*, *nnrR*, and *narR*, were thought to be responsible as primary effectors to oxygen, NO, and NO_3_^−^/NO_2_^−^ [13,44,45]. Furthermore, when using nitrate as sole nitrogen source, both respiratory nitrate reductases and assimilatory nitrate reductases were conducted together through a corporate chaperone encoded by *narJ* in the *Paracoccus* denitrification strain [46]. However, though this study proved the aerobic nitrate removal was caused by the respiratory nitrate reductases under transcriptional results, the nitrate distribution mechanism in assimilation is still unclear. Therefore, the difference of response and remodeled rules in *Pesudomaonas balearica* with other aerobic denitrificans need to be further studied. 

#### 3.3.2. Heterotrophic Ammonium Removal Pathway

**Figure 4:**
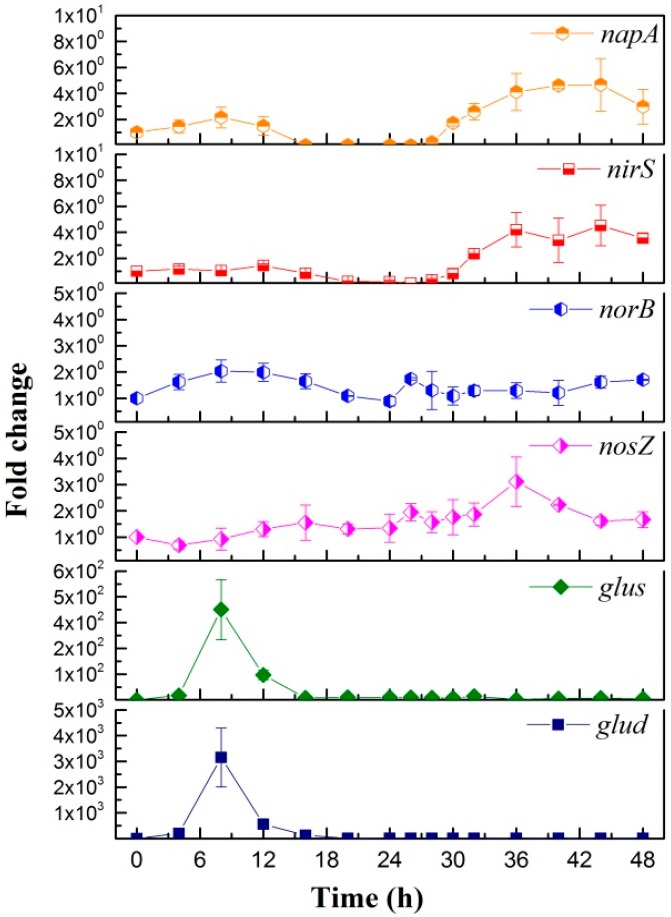
Denitrifying and glutamic acid related genes expression under heterotrophic ammonium removal of the strain RAD-17 during 48 h of incubation.

The transcriptional expression levels of the ammonium assimilation genes under aerobic condition of the strain RAD-17 are shown in Figure 4. In general, ammonium assimilation into different amino acids was the start for glutamate. Based on the KEGG nitrogen metabolism pathways, there are two major biosynthesis pathways of ammonium into L-glutamate, which involve glutamate dehydrogenase (1.4.1.2, 1.4.1.3, 1.4.1.4) as well as the glutamine synthetase (6.3.1.2) and glutamate synthase (1.4.1.13, 1.4.1.14, 1.4.7.1) [6]. In this study, an obvious up-regulation of *gluS* and *gluD* genes occurred during 4–16 h, which were approximately 420 and 3100-folds compared with the control sample, respectively. The ammonium concentrations also showed apparent consistency in this phase with a sharp decrease (Figure 2C). A similar phenomenon was also reported in other aerobic denitrificans, like *Pseudomonas stutzeri* T13 [22], *Acinetobacter sp.* HA2 [21], *Paracoccus versutus* LYM [36], and *Klebsiella sp* [47]. Therefore, this indicated that a certain assimilation pathway is performed in the strain RAD-17 when using ammonium as the sole nitrogen source.

On the other hand, the respiratory nitrate reductases-related genes of *napA*, *nirS*, *norB*, and *nosZ* did not show obvious enhanced expression during 0–24 hours, which indicated that ammonium should not be a direct inducer for aerobic denitrification. It should be noted that a slight nitrate accumulation occurred between 24–48 hours (Figure 2C), which also caused inconspicuous increase in the expression of nitrate reductases genes (Figure 4). In a previous study, ammonium translated into nitrate in aerobic denitrifications through hydroxylamine related genes [47]. However, we searched the whole genome of the strain RAD-17 (data not shown), but no hydroxylamine genes were found, which indicated that another potential pathway existed for the nitrate production and further denitrification. Thus, the results indicated that a novel pathway was existed for ammonia change to nitrogen-gas that none study was reported before. Hence, a hypothesis was proposed that the strain RAD-17 was inclined to reserve nitrate under ammonium feast condition. Basically, ammonium is a more available nutrient for microbes, while nitrate is a selective substance. Therefore, this might be a characteristic of the strain RAD-17 to fit a wider niche, but the mechanism needs further study.

### 3.4. Bioaugmentation Performance Evaluation

**Figure 5:**
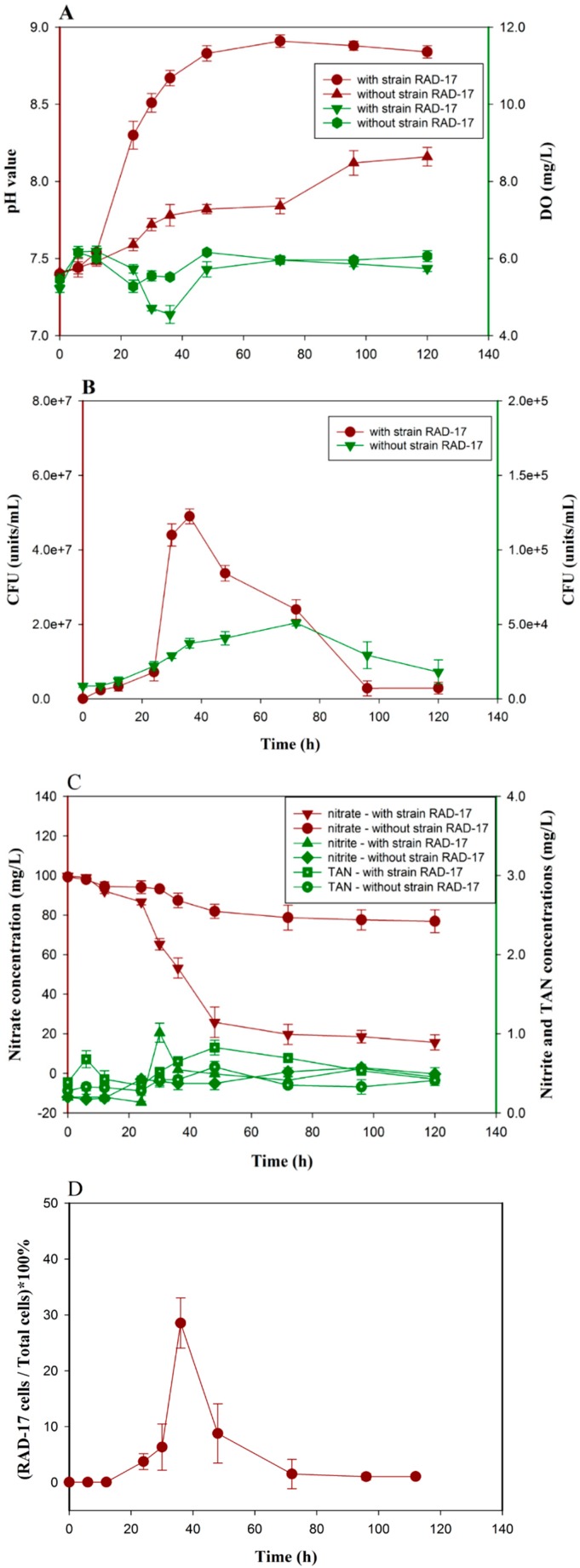
The bioaugmentation performance of the RAD-17 strain in actual recirculating aquaculture system (RAS) effluent. (**A**) pH and DO values; (**B**) maximum potential denitrifying strains numbers; (**C**) inorganic nitrogen concentrations; (**D**) strain RAD-17 abundance. Data shown are means ± SD (error bars) from three replicates.

The bioaugmentation performance by adding the strain RAD-17 into actual RAS effluent is shown in Figure 5. In general, the inoculated groups have higher nitrate removal efficiency compared with control groups (84.22% vs. 22.54%). The nitrate concentrations decreased from initially 99.27 ± 0.43 mg/L to 15.66 ± 3.85 mg/L or 76.89 ± 5.79 mg/L, respectively. In addition, the inoculated groups also gained relative higher pH values and lower DO values (Figure 5A,C), since heterotrophic denitrification was an alkalinity produced process [48]. Meanwhile, the strain RAD-17 also showed obvious preponderant cell abundance in wastewater, which revealed its potentially strong fitness in environment. The maximum denitrifying strains of 4.9 × 10^7^ ± 2.0 × 10^6^ cells/mL was found after 36 h of incubation by inoculated the strain RAD-17, while only 5.1 × 10^5^ ± 2.0 × 10^6^ cells/mL was gained in control groups (Figure 5B). It should be also noted that after 50 h, the relative abundance of strain RAD-17 was decrease, and the reasons might be related to substance insufficient or other strain competitive. Based on the qPCR detection, the strain RAD-17 occupied a peak ratio of 28.5 ± 4.5% in total strain cell numbers (Figure 5D). No significant amplification of the *napA* gene was detected in control groups by gel electrophores analysis (Figure S3). Bioaugmentation was a convinced technology to improve bioremediation system performance [49]. Therefore, our results provided clear evidence that the strain RAD-17 can function as efficient nitrate removal in RAS effluent treatment. 

### 3.5. Research Prospective

**Figure 6:**
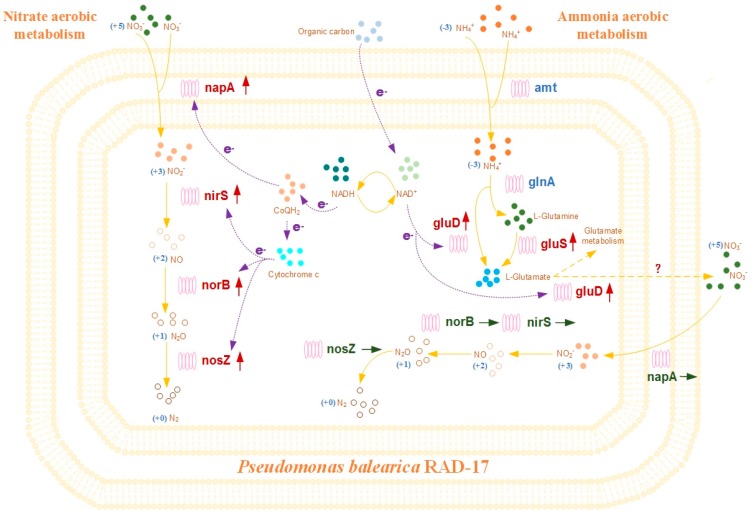
Proposed model for aerobic nitrogen removal mechanisms of the *Pseudomonas balearica* strain RAD-17. Red arrow means gene up-regulation; green arrow means inconspicuous gene regulation.

A proposed model for aerobic nitrogen removal mechanisms of the strain RAD-17 is shown in Figure 6, which indicated the convinced substance utilization and electron transportation pathways. The nitrate reductases that encoded at least five denitrification relative genes, *napA*, *napB*, *napC*, *napD*, and *napE*, were found to orderly arrange in clusters by genome annotation of the strain RAD-17, which should support the high nitrate removal efficiency. Respiratory nitrate reductases were carried out by *napA* primarily for aerobic denitrification, as well as the cell assimilation were a predominant approach started from *gluS* and *gluD* genes for ammonium metabolism. Taking into consideration the halotolerant characteristic and bioaugmentation performance, the above abilities support that the strain RAD-17 owns width ecological niche, and thus might have stronger fitness in the application.

However, the current phenomenon also indicated that deeper insightful research should be done for characterizing the strain RAD-17. The express mechanism of respiratory nitrate reductases and assimilatory nitrate reductases should be clearer under aerobic or alternate aerobic/anoxic conditions. The DO shifts commonly existed in temporal or spatial difference in practice to reveal potential chaperone, and transcriptional activators were in favor of setting unequivocal operated parameters in wastewater treatment project. Furthermore, the novel pathways responsible for the ammonia translated to nitrogen-gas should be illuminated clearly. 

Finally, the practical purpose is to use the strain RAD-17 for bioaugmentation to improve denitrification performance in RAS effluent. The whole cell immobilization and integrated with packing carrier should be considered. In addition, the function and ecological fitness of this individual species with other microbes that relate with QS (quorum sensing) regulation should be further clarified, and the relevant control methodologies still need further study.

## 4. Conclusions

An aerobic denitrification *Pseudomonas balearica* strain RAD-17 showed efficient nitrogen removal performance with average NO_3_^−^-N, NO_2_^−^-N, and TAN removal rates of 6.22 mg·L^−1^·h^−1^, 6.30 mg·L^−1^·h^−1^, and 1.56 mg·L^−1^·h^−1^, respectively. The transcriptional results proved that aerobic nitrogen metabolic pathways were performed in respiratory nitrate reductases (*napA*, *nirS*, *norB,* and *nosZ*) for nitrate removal, or cell assimilation (*gluS* and *gluD*) for ammonium utilization. In addition, the bioaugmentation performance by the strain RAD-17 achieved maximum cell abundance of 28.5 ± 4.5% in total environmental cell numbers, as well as obvious higher denitrification performance than in the no inoculum group (84.22% vs. 22.54%).

## Figures and Tables

**Table 1:** All the primers used in this study for *Pseudomonas balearica* strain RAD-17.

Gene	Primer Sequences (5′-3′)	References
16S rRNA	F: CCTACGGGAGGCAGCAG	This study
	R: ATTACCGCGGCTGCTGG	
*glu*D	F: GCTATCGCATCCAGATGAAC	This study
	R: CATCACTTCGTTGTCGCTC	
*glu*S	F: CGCAACATCTTCTCCAACCC	This study
	R: TTCTCCTCACCCCATTCGAC	
*nap*A	F: TTCATGGCCTGCTGTACCTG	This study
	R: TCATCCTGGCGCAATCGAAC	
*nir*S	F: TGGAAAGCCAGATGCAGCAC	This study
	R: ACGCTCCTTGACGAAGTGGATG	
*nor*B	F: TTCTACAACCCCGAGAACC	This study
	R: GCAATGATGACGTACAGCC	
*nos*Z	F: CAACATCGACCAGATCGAAG	This study
	R: TGCAGTAGTACCAGTGCAG	

**Table 2:** Of the RAD-17 strain determined by analytical profile index of Gram-negative with non-Enterobacteriaceae (API 20NE) tests.

API 20NE Results	Strain RAD-17
Oxidase test Nitrate reduction	− +
Arginine dihydrolase	−
Urease	−
β-Glucosidase	−
Protease	−
β-Galactosidase	−
Assimilation of	
Glucose	+
Arabinose	−
Mannose	−
Mannitol	−
*N*-acetyl-glucosamine	−
Maltose	+
Gluconate	+
Capric acid	+
Adipic acid	−
Malic acid	+
Citric acid	+
Phenylacetic acid	−

**+** Positive; **−** Negative.

**Table 3:** Balance analysis for the strain RAD-17 under aerobic denitrification.

Substance	Initial TN (mg/L)	Final Nitrogen (mg/L)				Intracellular N	N Loss (%)
		NO_3_^−^-N	NO_2_^−^-N	NH_4_^+^-N	Organic-N		
Nitrate	30.56 ± 0.02	0.14 ± 0.03	0.05 ± 0.02	0.17 ± 0.06	0.15 ± 0.04	3.23 ± 0.34	87.76

Note: N loss = (Initial TN–Final N–Intracellular N)/Initial TN * 100%.

**Table 4:** Varied single factors on the aerobic denitrification performance of strain RAD-17 after 48 h incubation.

Factor	Variations	Initial Nitrate (mg/L)	Final Nitrate (mg/L)	Final Nitrite (mg/L)	Final TAN (mg/L)	Growth (OD_600_)
**C/N** **ratios**	2	295.48 ± 0.60	149.00 ± 2.87	50.21 ± 1.52	8.61 ± 1.19	0.86 ± 0.16
	5	298.42 ± 0.26	0.92 ± 0.80	0.36 ± 0.02	1.88 ± 0.04	1.17 ± 0.10
	10	299.59 ± 0.37	8.17 ± 1.82	0.34 ± 0.01	1.19 ± 0.03	1.43 ± 0.03
	15	301.51 ± 0.71	8.55 ± 5.10	0.30 ± 0.00	1.02 ± 0.03	1.04 ± 0.25
	20	300.09 ± 0.71	23.41 ± 9.78	0.64 ± 0.02	0.95 ± 0.00	1.09 ± 0.14
**NaCl** **(‰)**	0	302.47 ± 0.19	3.14 ± 2.00	0.34 ± 0.01	0.26 ± 0.08	1.73 ± 0.08
	2.5	299.78 ± 0.56	6.52 ± 1.37	0.42 ± 0.03	0.30 ± 0.05	1.50 ± 0.06
	5	301.29 ± 0.24	9.37 ± 5.57	0.27 ± 0.03	1.04 ± 0.08	1.30 ± 0.15
	15	300.36 ± 0.17	8.11 ± 4.29	0.23 ± 0.07	1.05 ± 0.02	1.40 ± 0.35
	25	301.46 ± 0.33	9.04 ± 4.11	0.25 ± 0.04	1.14 ± 0.06	1.66 ± 0.24
**Carbon** **source**	Fructose	299.61 ± 0.22	205.59 ± 8.50	5.71 ± 4.08	29.13 ± 12.13	0.27 ± 0.03
	NaAC	303.14 ± 0.11	2.39 ± 1.11	0.87 ± 0.02	3.34 ± 0.33	1.99 ± 0.11
	Lactin	298.45 ± 0.27	296.90 ± 2.59	0.36 ± 0.03	–	0.69 ± 0.15
	Glucose	300.12 ± 0.09	4.71 ± 1.64	1.03 ± 0.03	2.23 ± 0.65	1.93 ± 0.05
	Na-citrate	301.09 ± 0.14	0.57 ± 0.27	0.51 ± 0.04	0.10 ± 0.09	1.86 ± 0.11
**Rotation** **Speed** **(rpm)**	0	304.56 ± 0.15	19.16 ± 6.26	0.35 ± 0.11	1.01 ± 0.40	1.37 ± 0.06
	50	302.42 ± 0.31	36.57 ± 4.53	1.07 ± 0.25	1.68 ± 0.57	0.76 ± 0.05
	100	301.77 ± 0.17	116.52 ± 9.91	13.74 ± 0.20	0.90 ± 0.65	1.07 ± 0.11
	150	299.46 ± 0.20	8.99 ± 1.33	0.54 ± 0.04	1.71 ± 0.29	2.00 ± 0.07
	200	301.26 ± 0.05	2.59 ± 0.94	0.53 ± 0.16	1.86 ± 0.93	2.19 ± 0.06
**Temperature** **(°C)**	5	299.76 ± 0.16	298.57 ± 1.50	–	0.45 ± 0.29	0.74 ± 0.06
	15	298.31 ± 0.09	7.79 ± 0.91	0.62 ± 0.06	1.63 ± 1.42	1.89 ± 0.18
	25	300.55 ± 0.11	2.28 ± 2.21	0.41 ± 0.07	2.49 ± 0.10	2.03 ± 0.25
	40	300.98 ± 0.17	18.51 ± 5.32	0.60 ± 0.18	2.50 ± 1.20	1.70 ± 0.15

## Supplementary Materials

The following are available online at https://www.mdpi.com/2076-2607/8/1/72/s1, Figure S1: Scanning electron microscope micrograph of Pseudomonas balearica RAD-17; Figure S2: The specificity evaluation of PCR amplification assay. The specific DNA bands were detected by agarose gel electrophoresis, lane 1-7 represent the amplified products of 16S rDNA, gluD, gluS, napA, nirS, norB and nosZ, respectively; Figure S3: The agarose gel electrophoresis of the qRT-PCR amplification product of napA gene (A) and 16S rDNA genes (B) in one of the no-inoculum treatments. Lane 1-8 represent the time point of eight samples from 0 hour to 72 hours (including 0, 6, 12, 24, 30, 36, 48, 72 hours).M, marker; CK, positive control.

## References

[1] Winkler M.K.H., Straka L. (2019). New directions in biological nitrogen removal and recovery from wastewater. Curr. Opin. Biotechnol..

[2] Lu H., Chandran K., Stensel D. (2014). Microbial ecology of denitrification in biological wastewater treatment. Water Res..

[3] Zhu S.-M., Deng Y.-L., Ruan Y.-J., Guo X.-S., Shi M.-M., Shen J.-Z. (2015). Biological denitrification using poly (butylene succinate) as carbon source and biofilm carrier for recirculating aquaculture system effluent treatment. Bioresour. Technol..

[4] Wang J., Chu L. (2016). Biological nitrate removal from water and wastewater by solid-phase denitrification process. Biotechnol. Adv..

[5] Qiu T., Liu L., Gao M., Zhang L., Tursun H., Wang X. (2016). Effects of solid-phase denitrification on the nitrate removal and bacterial community structure in recirculating aquaculture system. Biodegradation.

[6] Ruan Y.-J., Deng Y.-L., Guo X.-S., Timmons M.B., Lu H.-F., Han Z.-Y., Ye Z.-Y., Shi M.-M., Zhu S.-M. (2016). Simultaneous ammonium and nitrate removal in an airlift reactor using poly (butylene succinate) as carbon source and biofilm carrier. Bioresour. Technol..

[7] Sun H., Yang Z., Wei C., Wu W. (2018). Nitrogen removal performance and functional genes distribution patterns in solid-phase denitrification sub-surface constructed wetland with micro aeration. Bioresour. Technol..

[8] Wu W., Yang F., Yang L. (2012). Biological denitrification with a novel biodegradable polymer as carbon source and biofilm carrier. Bioresour. Technol..

[9] Li P., Zuo J., Wang Y., Zhao J., Tang L., Li Z. (2016). Tertiary nitrogen removal for municipal wastewater using a solid-phase denitrifying biofilter with polycaprolactone as the carbon source and filtration medium. Water Res..

[10] Giblin A.E., Tobias C.R., Song B., Weston N., Banta G.T., Rivera-Monroy V.H. (2013). The importance of dissimilatory nitrate reduction to ammonium (DNRA) in the nitrogen cycle of coastal ecosystems. Oceanography.

[11] Gentry T., Rensing C., Pepper I.A.N. (2004). New approaches for bioaugmentation as a remediation technology. Crit. Rev. Environ. Sci. Technol..

[12] Zhang S., Sun X., Wang X., Qiu T., Gao M., Sun Y., Cheng S., Zhang Q. (2018). Bioaugmentation with *Diaphorobacterpolyhydroxybutyrativorans* to enhance nitrate removal in a poly (3-hydroxybutyrate-co-3-hydroxyvalerate)-supported denitrification reactor. Bioresour. Technol..

[13] Zumft W.G. (1997). Cell biology and molecular basis of denitrification. Microbiol. Mol. Biol. Rev..

[14] Lv P.-L., Shi L.-D., Wang Z., Rittmann B., Zhao H.-P. (2019). Methane oxidation coupled to perchlorate reduction in a membrane biofilm batch reactor. Sci. Total Environ..

[15] Körner H., Zumft W.G. (1989). Expression of denitrification enzymes in response to the dissolved oxygen level and respiratory substrate in continuous culture of *Pseudomonas stutzeri*. Appl. Environ. Microbiol..

[16] Ji B., Yang K., Zhu L., Jiang Y., Wang H., Zhou J., Zhang H. (2015). Aerobic denitrification: A review of important advances of the last 30 years. Biotechnol. Bioprocess. Eng..

[17] Morozkina E.V., Zvyagilskaya R.A. (2007). Nitrate reductases: Structure, functions, and effect of stress factors. Biochemistry.

[18] Chen J., Strous M. (2013). Denitrification and aerobic respiration, hybrid electron transport chains and co-evolution. Biochim. Biophys. Acta-Bioenerg..

[19] Deng Y., Ruan Y., Ma B., Timmons M.B., Lu H., Xu X., Zhao H., Yin X. (2019). Multi-omics analysis reveals niche and fitness differences in typical denitrification microbial aggregations. Environ. Int..

[20] Zhang J., Wu P., Hao B., Yu Z. (2011). Heterotrophic nitrification and aerobic denitrification by the bacterium *Pseudomonas stutzeri*YZN-001. Bioresour. Technol..

[21] Yao S., Ni J., Ma T., Li C. (2013). Heterotrophic nitrification and aerobic denitrification at low temperature by a newly isolated bacterium, *Acinetobacter sp*. HA2. Bioresour. Technol..

[22] Sun Y., Feng L., Li A., Zhang X., Yang J., Ma F. (2017). Ammonium assimilation: An important accessory during aerobic denitrification of *Pseudomonas stutzeri*T13. Bioresour. Technol..

[23] Kong D., Li W., Deng Y., Ruan Y., Chen G., Yu J., Lin F. (2018). Denitrification-potential evaluation and nitrate-removal-pathway analysis of aerobic denitrifier strain *Marinobacterhydrocarbonoclasticus* RAD-2. Water.

[24] Ji B., Yang K., Wang H., Zhou J., Zhang H. (2015). Aerobic denitrification by *Pseudomonas stutzeri*C3 incapable of heterotrophic nitrification. Bioprocess Biosyst. Eng..

[25] Sun Y., Li A., Zhang X., Ma F. (2015). Regulation of dissolved oxygen from accumulated nitrite during the heterotrophic nitrification and aerobic denitrification of *Pseudomonas stutzeri*T13. Appl. Microbiol. Biotechnol..

[26] Chen J., Gu S., Hao H., Chen J. (2016). Characteristics and metabolic pathway of *Alcaligenes sp*. TB for simultaneous heterotrophic nitrification-aerobic denitrification. Appl. Microbiol. Biotechnol..

[27] Deng Y.L., Ruan Y.J., Zhu S.M., Guo X.S., Han Z.Y., Ye Z.Y., Liu G., Shi M.M. (2017). The impact of DO and salinity on microbial community in poly(butylene succinate) denitrification reactors for recirculating aquaculture system wastewater treatment. AMB Express..

[28] Garthright W.E., Blodgett R.J. (2003). FDA’s preferred MPN methods for standard, large or unusual tests, with a spreadsheet. Food Microbiol..

[29] SEPA (2002). Water and Wastewater Monitoring Methods.

[30] Huang T., Guo L., Zhang H., Su J., Wen G., Zhang K. (2015). Nitrogen-removal efficiency of a novel aerobic denitrifying bacterium, *Pseudomonas stutzeri* strain ZF31, isolated from a drinking-water reservoir. Bioresour. Technol..

[31] Gui M., Chen Q., Ni J. (2017). Effect of sulfamethoxazole on aerobic denitrification by strain *Pseudomonas stutzeri* PCN-1. Bioresour. Technol..

[32] He T., Li Z., Sun Q., Xu Y., Ye Q. (2016). Heterotrophic nitrification and aerobic denitrification by *Pseudomonas tolaasii* Y-11 without nitrite accumulation during nitrogen conversion. Bioresour. Technol..

[33] Li C., Yang J., Wang X., Wang E., Li B., He R., Yuan H. (2015). Removal of nitrogen by heterotrophic nitrification-aerobic denitrification of a phosphate accumulating bacterium *Pseudomonas stutzeri*YG-24. Bioresour. Technol..

[34] Jin R., Liu T., Liu G., Zhou J., Huang J., Wang A. (2015). Simultaneous heterotrophic nitrification and aerobic denitrification by the marine origin bacterium *Pseudomonas sp*. ADN-42. Appl. Biochem. Biotechnol..

[35] Zhang H., Zhao Z., Chen S., Kang P., Wang Y., Feng J., Jia J., Yan M., Wang Y., Xu L. (2018). *Paracoccusversutus*KS293 adaptation to aerobic and anaerobic denitrification: Insights from nitrogen removal, functional gene abundance, and proteomic profiling analysis. Bioresour. Technol..

[36] Shi Z., Zhang Y., Zhou J., Chen M., Wang X. (2013). Biological removal of nitrate and ammonium under aerobic atmosphere by *Paracoccusversutus* LYM. Bioresour. Technol..

[37] Borrero-de Acuña J.M., Timmis K.N., Jahn M., Jahn D. (2017). Protein complex formation during denitrification by *Pseudomonas aeruginosa*. Microb. Biotechnol..

[38] Zheng H.-Y., Liu Y., Gao X.-Y., Ai G.-M., Miao L.-L., Liu Z.-P. (2012). Characterization of a marine origin aerobic nitrifying–denitrifying bacterium. J. Biosci. Bioeng..

[39] Zhang Q.-L., Liu Y., Ai G.-M., Miao L.-L., Zheng H.-Y., Liu Z.-P. (2012). The characteristics of a novel heterotrophic nitrification-aerobic denitrification bacterium, *Bacillus methylotrophicus* strain L7. Bioresour. Technol..

[40] Fernandez-Linares L., Acquaviva M., Bertrand J.-C., Gauthier M. (1996). Effect of sodium chloride concentration on growth and degradation of eicosane by the marine halotolerant bacterium *Marinobacterhydrocarbonoclasticus*. Syst. Appl. Microbiol..

[41] Gouesbet G., Blanco C., Hamelin J., Bernard T. (1992). Osmotic adjustment in Brevibacteriumammoniumgenes: Pipecolic acid accumulation at elevated osmolalities. Microbiology.

[42] Liu Y., Ai G.-M., Miao L.-L., Liu Z.-P. (2016). *Marinobacter strain* NNA5, a newly isolated and highly efficient aerobic denitrifier with zero N_2_O emission. Bioresour. Technol..

[43] Saleh-Lakha S., Shannon K.E., Henderson S.L., Goyer C., Trevors J.T., Zebarth B.J., Burton D.L. (2009). Effect of pH and temperature on denitrification gene expression and activity in *Pseudomonas mandelii*. Appl. Environ. Microbiol..

[44] Giannopoulos G., Sullivan M.J., Hartop K.R., Rowley G., Gates A.J., Watmough N.J., Richardson D.J. (2017). Tuning the modular *Paracoccus* denitrificans respirome to adapt from aerobic respiration to anaerobic denitrification. Environ. Microbiol..

[45] Baker S.C., Ferguson S.J., Ludwig B., Page M.D., Richter O.-M.H., van Spanning R.J.M. (1998). Molecular genetics of the genus *Paracoccus*: Metabolically versatile bacteria with bioenergetic flexibility. Microbiol. Mol. Biol. Rev..

[46] Pinchbeck B.J., Soriano-Laguna M.J., Sullivan M.J., Luque-Almagro V.M., Rowley G., Ferguson S.J., Roldán M.D., Richardson D.J., Gates A.J. (2019). A dual functional redox enzyme maturation protein for respiratory and assimilatory nitrate reductases in bacteria. Mol. Microbiol..

[47] Jin P., Chen Y., Yao R., Zheng Z., Du Q. (2019). New insight into the nitrogen metabolism of simultaneous heterotrophic nitrification-aerobic denitrification bacterium in mRNA expression. J. Hazard. Mater..

[48] Kim E.-W., Bae J.-H. (2000). Alkalinity requirements and the possibility of simultaneous heterotrophic denitrification during sulfur-utilizing autotrophic denitrification. Water Sci. Technol..

[49] Wang R., Zheng P., Zhang M., Zhao H.-P., Ji J.-Y., Zhou X.-X., Li W. (2015). Bioaugmentation of nitrate-dependent anaerobic ferrous oxidation by heterotrophic denitrifying sludge addition: A promising way for promotion of chemoautotrophic denitrification. Bioresour. Technol..

