# Supplementary material for: Nitrogen Removal Performance and Metabolic Pathways Analysis of a Novel Aerobic Denitrifying Halotolerant Pseudomonas balearica Strain RAD-17
Source: Microorganisms. 2020 Jan 2;8(1):72. doi: 10.3390/microorganisms8010072 (PMC7022906; doi:10.3390/microorganisms8010072)
Supplement: Supplementary file 1 [file microorganisms-08-00072-s001.pdf]

Supplementary materials

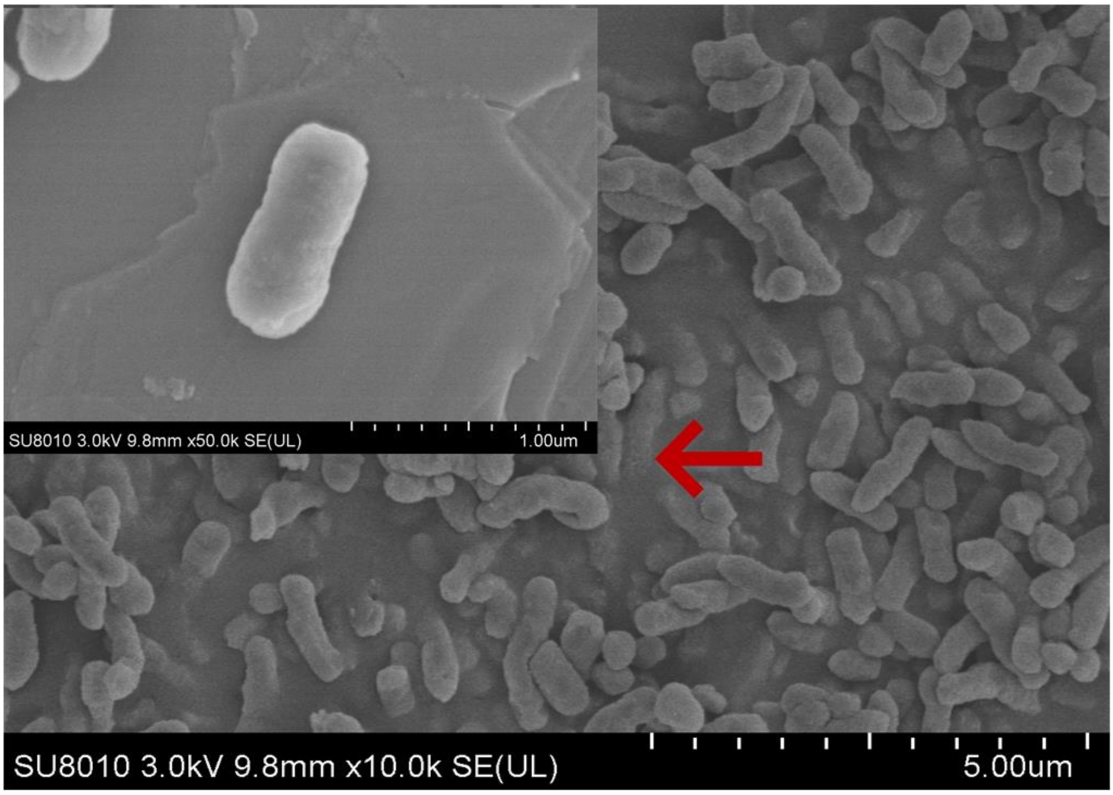

Figure S1. Scanning electron microscope micrograph of *Pseudomonas balearica* RAD-17.

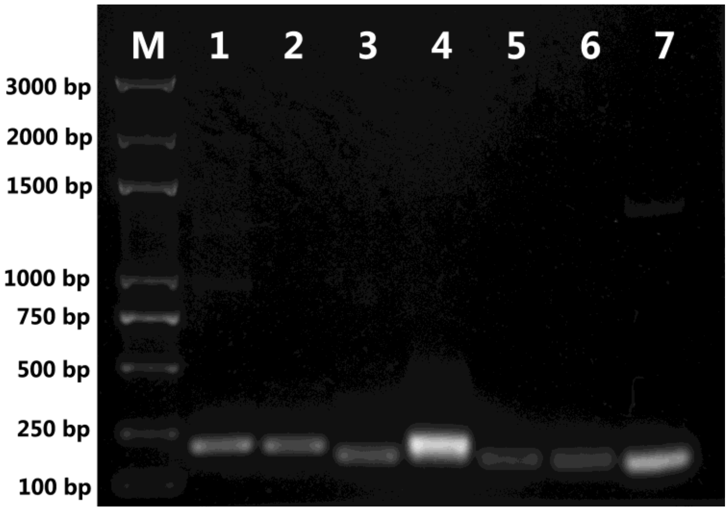

Figure S2. The specificity evaluation of PCR amplification assay. The specific DNA bands were detected by agarose gel electrophoresis, lane 1-7 represent the amplified products of 16S rDNA, *glu*D, *glu*S, *nap*A, *nir*S, *nor*B and *nos*Z, respectively.

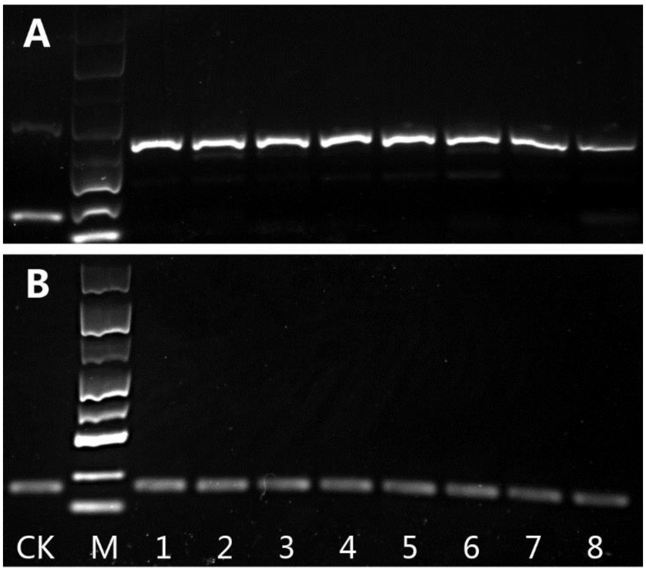

Figure S3. The agarose gel electrophoresis of the qRT-PCR amplification product of *napA* gene (A) and 16S rDNA genes (B) in one of the no-inoculum treatments. Lane 1-8 represent the time point of eight samples from 0 hour to 72 hours (including 0, 6, 12, 24, 30, 36, 48, 72 hours).M, marker; CK, positive control.
